# Investigating the emigration intention of health care workers: A cross‐sectional study

**DOI:** 10.1002/nop2.2170

**Published:** 2024-05-21

**Authors:** Oluwaseun Abdulganiyu Badru, Tunde Adeyemo Alabi, Samuel Sijibomi Okerinde, Muhammad Auwal Kabir, Aisha Abdulrazaq, Oluwafemi Atanda Adeagbo, Fatai Adesina Badru

**Affiliations:** ^1^ Department of Community and Behavioral Health University of Iowa Iowa City Iowa USA; ^2^ Usmanu Danfodiyo University Teaching Hospital Sokoto Nigeria; ^3^ Department of Sociology University of Lagos Lagos Nigeria; ^4^ Department of Sociology University of Cape Town Cape Town South Africa; ^5^ Department of Community Health and Primary Care Lagos University Teaching Hospital Lagos Nigeria; ^6^ Department of Physiotherapy Bayero University Kano Nigeria; ^7^ Malaria Consortium Sokoto Nigeria; ^8^ Department of Sociology University of Johannesburg Johannesburg South Africa; ^9^ Department of Social Work University of Lagos Lagos Nigeria

**Keywords:** burnout, emigration intention, health care workers, job satisfaction, Nigeria

## Abstract

**Aims:**

To (1) explore the intramigration experience of HCWs within Nigeria, (2) explore the migration intention of health care workers (HCWs) in Nigeria and (3) identify the predictors of migration intention among HCWs in Nigeria.

**Design:**

Cross‐sectional study.

**Methods:**

The online survey was used to collect data from 513 HCWs in Nigeria between May and June 2023. Crude and adjusted logistic regression were used to identify factors associated with emigration intention. Analyses were performed on SPSS version 26 at a 95% confidence interval.

**Results:**

The study found that 34.4% had intramigration experience, and the rate of intention to emigrate to work in another country was 80.1%. The United Kingdom was the most preferred destination (109 HCWs), followed by Canada (92 HCWs) and the United States (82 HCWs). At the multivariate level, emigration intention was associated with the experience of burnout and duration of practice as a HCW. Nurses had higher emigration intentions than medical doctors.

**Conclusions:**

Many HCWs in Nigeria appear to have emigration intent, and nurses are more likely to be willing to migrate than doctors. The Nigerian government may want to explore strategies to reverse the emigration intent of the HCWs in Nigeria.

## 1 INTRODUCTION

Migration relates to the movement of a person from the usual place of residence to another place, within or across international borders, temporarily or permanently, and usually for different reasons (IOM, 2023). However, the common movement pattern is from a low‐resource area to a high‐resource area (e.g., rural to urban area) or a low‐income to a high‐income country (Amorha et al., 2022). This pattern of migration may result in a brain drain of the low‐resource area and brain gain to the high‐resource area (Anduaga‐Beramendi et al., 2019). For instance, 30% of pharmacists in Canada are trained outside, similar to the 10% in Australia, which is a sign of brain gain at the expense of less low‐income countries (Amorha et al., 2022; Jackson et al., 2021). Migration of health care workers (HCWs) is an ongoing public health concern due to the negative impact on the health workforce, health indexes of the sending countries and its impact on widening the existing socioeconomic inequalities (Amorha et al., 2022). Regrettably, the Caribbean region and sub‐Saharan Africa (SSA) are the worst hit by HCWs’ migration, and Nigeria is no exception (Anduaga‐Beramendi et al., 2019; Onah et al., 2022). A past study reported that India, the Philippines, China, Malaysia, Pakistan, Colombia and Egypt were the largest exporters of HCWs to high‐income countries (Nair & Webster, 2013). Nigeria has joined the rank of the highest donor of HCWs to the high‐income countries, complicated by the exhaustion and job dissatisfaction following the COVID‐19 pandemic (Lawal et al., 2022).

## 2 BACKGROUND

As of 2021, there were 84,277 doctors and 3.95 doctors per 10,000 population in Nigeria, significantly less than the 1:400–600 doctor‐population ratio recommended by the World Health Organization (WHO, 2023). The trend of Nigerian HCWs migrating to other countries is alarming and disturbing (Onah et al., 2022). As of October 2022, Nigeria had one doctor for about 10,000 patients (Adejoro, 2022). Despite the shortage of critical HCWs in Nigeria, many of them are emigrating. It was reported that the United Kingdom licensed 142 doctors from Nigeria in early 2023 (Adejoro, 2023). Furthermore, over 500 medical and dental consultants had migrated to other countries as of 2022 (ibid). Realizing the emigration of HCWs in Nigeria, the National Assembly attempted to make a law that would mandate medical doctors to work for 5 years in Nigeria after completing their education before emigrating (The Guardian, 2023).

There is evidence that more HCWs are intending or planning to migrate, and a local slogan for emigration has been coined “japa” or “japa syndrome” among Nigerians (Alabi & Olajide, 2023). A recent survey showed that more than half (57.4%) of the 244 doctors surveyed in a Nigerian teaching hospital located in Oyo State (Southwestern region) had migration intentions and more than one‐third (34.8%) had made attempts at emigrating (Adebayo & Akinyemi, 2022). Another teaching hospital‐based study conducted in the Ekiti State (Southwestern region), Nigeria, reported a 72.4% prevalence of migration intention among resident doctors (Akinwumi et al., 2022). An online survey across all 36 states in Nigeria plus the Federal Capital Territory (FCT) found that 43.9% of 913 doctors had migration intention (Onah et al., 2022).

The phrase push and pull factors often describe the reasons for wanting to migrate (see Adovor et al., 2021; Öncü et al., 2021; Pinto da Costa et al., 2021). Push factors are often perceived as negative factors by HCWs, while pull factors are ascribed to positive factors. This does not necessarily imply that the push factor is the opposite of the pull factor, nor does it mean that the perceived positivity of the pull factor negates that of the push factor (Nguyen & Wood, 2019). Nguyen and Wood (2019) argued that the accuracy of perceived push and pull factors differs, “A migrant is more likely to perceive push factors accurately than pull factors, given that the point of origin is more familiar than the destination” (p. 3). Furthermore, pull factors can lead to what Amorha et al. (2022) called “brain waste” (p. 1), which is a situation whereby skilled HCWs end up working as unskilled labour in non‐health sectors. This may be considered a waste because such migration leaves the home country short of the needed health care workforce, and the HCWs are not utilized as the needed health force in the host country.

In the context of HCWs, push factors are conditions in the home country that make HCWs want to migrate to another country (Anduaga‐Beramendi et al., 2019; Nguyen & Wood, 2019). The common push factors in Nigeria and elsewhere include poor salaries, infrastructure, insecurity and political instability (Hashish & Ashour, 2020; Onah et al., 2022). On the other hand, pull factors are external features that entice HCWs to the host country (Adebayo & Akinyemi, 2022; Hashish & Ashour, 2020). These factors include good salary and benefits, career advancement opportunities and health‐system quality (Adebayo & Akinyemi, 2022; Hashish & Ashour, 2020).

Factors associated with migration intention among HCWs are well documented in the literature and are situated across different socioecological levels. Intrapersonal factors such as age, gender and poor socioeconomic status are reported predictors of migration intention globally (Anduaga‐Beramendi et al., 2019). Organizational or work‐related factors such as work experience, job satisfaction and burnout from excessive work have been identified as predictors of migration intention among HCWs (Dubas‐Jakóbczyk et al., 2020; Győrffy et al., 2018; Yakubu et al., 2023). Some of the mentioned factors have shown inconsistent predictive power across different studies. Specifically, intrapersonal factors such as religion, ethnicity, marital status, number of children and environmental factors such as region and place of residence are less common predictors of migration in the literature, as reported in earlier studies from Nigeria (Adebayo & Akinyemi, 2022; Akinwumi et al., 2022; Onah et al., 2022; Yakubu et al., 2023). Burnout involves physical and mental exhaustion from social and work‐related stress and often develops over a long period (Sipos et al., 2023). Burnout from excess workload has been singled out as a driver of migration, and the increasing job dissatisfaction associated with burnout has compounded why HCWs want to leave their country (Győrffy et al., 2018; Ogboghodo & Edema, 2020). Lack of job satisfaction significantly impacts HCWs' effectiveness and efficiency (Sipos et al., 2017).

There are several studies on migration intentions among HCWs globally and in Nigeria. Yet, there are gaps to be filled in relation to HCWs’ migration in Nigeria. First, empirical studies on HCWs' migration intention in Nigeria have largely focused on doctors (Adebayo & Akinyemi, 2022; Akinwumi et al., 2022; Onah et al., 2022). However, a recent study included nurses and pharmacists in their analysis and found that they were more likely to be willing to migrate than doctors (Yakubu et al., 2023), suggesting that other medical professionals are increasingly considering migrating to other countries and should be included in HCW migration studies. In their mixed‐method study, Yakubu et al. (2023) excluded HCWs such as podiatrists and physiotherapists because they are less often mentioned in the literature, missing the opportunity to beam light on the emigration intention of the less commonly surveyed HCWs in Nigeria. This is a crucial gap that this present study aims to bridge because it is urgently important to quantify the migration intention of all skilled HCWs in light of the exponential HCWs’ emigration currently being witnessed in Nigeria.

Second, most studies on migration intention among HCWs have emerged from Southern Nigeria. The neglected Northern region has more states than the Southern region (19 vs. 17), excluding the FCT. Northern Nigeria has been previously described as a conservative region, which suggests that the region will relatively experience limited emigration compared to its cosmopolitan Southern region (Alabi et al., 2020; Alabi & Badru, 2021). The conservative lifestyle of the North may explain why migration experts have not investigated the emigration intent of HCWs in the region. Although Yakubu et al. (2023) recently assessed the migration intention of HCWs across the six geopolitical zones, including the Northern regions, many earlier migration studies did not consider Northern Nigeria (see Ajayi et al., 2022; Akinwumi et al., 2022; Onah et al., 2022). In a recent secondary data analysis of north–south differences in emigration intention in Nigeria, Alabi and Olajide (2023) found that the south had higher emigration intention than the north; the former prefers North America and Europe, while the latter prefers another African country. This testifies to the differences between the northern and southern regions (Alabi, 2023; Alabi & Badru, 2021). However, the study did not specifically focus on HCWs; it analysed secondary data from 1600 Nigerian adults.

The difference in lifestyle and socio‐behavioural patterns may have influenced HCWs' choice of where to live and work. This study hypothesizes that HCWs in the North will prefer host countries with similar cultures, religious affiliations or geographical ties, such as Saudi Arabia, Qatar or other African countries. However, the pattern of migration intention for HCWs in Southern Nigeria may differ; the cosmopolitanism of this region may tilt them towards the Western region, like the United States and United Kingdom. Therefore, it is important to investigate the intent of HCWs in both regions and their preferred destination.

Finally, many studies have reported reasons, often as push and pull factors, that stimulate migration intention in HCWs; many of these studies have described push–pull factors descriptively (Adebayo & Akinyemi, 2022; Hashish & Ashour, 2020; Onah et al., 2022). While this somewhat helps to quantify the reason HCWs might want to migrate, it does not show the true association with migration intention. This study will test the association between push and pull factors and migration intention analytically rather than descriptively. Such a finding may help scholars and policymakers identify which of the two factors (push or pull) is more debilitating to the Nigerian health sector. Such findings may lead to policy adjustment or formulation that will ensure a sticking factor (provisions that keep HCWs in their home country) and break stay factors (provisions that keep migrated HCWs from returning to their home country).

## 3 THE STUDY

### 3.1 Aims

This study aims to (1) explore the migration intention of health care workers HCWs in Nigeria, (2) explore intramigration experience of HCWs within Nigeria and (3) identify the predictors of migration intention among HCWs in Nigeria.

### 3.2 Design

The study adopted a cross‐sectional online survey in Nigeria.

### 3.3 Study area, study design and population

The population comprised HCWs who were practising in Nigeria at the time of the survey. A total of 636 people responded to the survey during the one‐month data collection. Sixty‐five were deleted as the respondents did not specify their profession, 46 because responses were from outside Nigeria, nine did not fill the outcome variables, and six records were empty. A total of 513 records were analysed, which gives a response rate of 80.7%. The study protocol was approved by the Sokoto State Ministry of Health (SKHREC/019/2023).

### 3.4 Data collection technique

Survey Monkey was used to develop the questionnaire. Survey Monkey helps to determine the location of the respondents, as we were interested in HCWs in Nigeria only. For convenience, the questionnaire was pretested among HCWs in a major hospital in Sokoto State (North West geopolitical zone) to assess suitability and detect ambiguity. Necessary corrections were effected before the final questionnaire was distributed. The final questionnaire was deployed on Survey Monkey to collect data between 26 May and 26 June 2023. All the responses were screened. The Internet Protocol (IP) address – collected by Survey – of each of the devices used for completing the response was checked. Any response from an IP address outside of Nigeria was deleted.

### 3.5 Sampling technique

HCWs were recruited online from the 36 Nigerian states and the FCT. Specifically, HCWs were recruited online; an advantage of an online recruitment method is assessing HCWs in hard‐to‐reach areas; it is a fast mode for data collection and suitable for researchers with minimal or no funding (Topp & Pawloski, 2002; Van Selm & Jankowski, 2006).

Popular online media such as WhatsApp, Telegram, Facebook and Twitter were leveraged to access HCWs. National and State chapters' social media groups for HCWs were leveraged to obtain data from HCWs in Nigeria, as previously done by Onah et al. (2022). Such groups include the Medical and Dental Consultants Association of Nigeria (MDCAN), the National Association of Resident Doctors (NARD) and the Joint Health Sector Union (JOHESU). The data collection period was 1 month. HCWs that reside outside Nigeria were excluded from the data analysis. The IP address captured by Survey Monkey helped identify HCWs who resided in Nigeria and those who had migrated.

### 3.6 Measures

#### 3.6.1 Migration intention

The main focus of this study is migration intention (i.e., willingness to migrate abroad), which was measured using a single‐item question. The HCWs were asked: “*Are you currently considering practicing your profession abroad?*”. The question was measured with four options: “definitely no,” “probably no,” “probably yes” and “definitely yes.” HCWs who selected “probably yes” and “definitely yes” were classified as having the intention to migrate, while those who selected “definitely no” and “probably no” were classified as not having the intention to migrate (Dubas‐Jakóbczyk et al., 2020). Furthermore, we asked the HCWs to name the countries they intend to migrate to. In addition, we asked about their intramigration experience within Nigeria, that is, from one of the six geopolitical zones to another. The variable was dichotomized into whether they had intramigration experience or not for inferential analysis.

#### 3.6.2 Push and pull factors

Push and pull questions were developed after an extensive literature review, including primary and systematic reviews (Adebayo & Akinyemi, 2022; Adovor et al., 2021; Benton et al., 2013; Dywili et al., 2013; Hashish & Ashour, 2020; Lawal et al., 2022; Mejía et al., 1979; Thapa & Shrestha, 2017). The push domain contains seven reasons for willingness to migrate abroad: personal, economic, political, professional development, work‐related factors, social and health‐system quality (Cronbach alpha: 0.886). The pull domain contains five reasons: economic, professional development, work‐related factors, social and health‐system quality (Cronbach alpha: 0.912). These factors were measured on a five‐point Likert scale (strongly disagree = 1 to strongly agree = 5).

#### 3.6.3 Burnout

Burnout was measured with the Maslach Burnout Inventory (MBI) Scale (Maslach et al., 1996). However, the validated single burnout item was used to assess burnout. HCWs were asked: “*I feel burned out from my work*”; the question was measured on a seven‐point Likert scale ranging from 0 = never to 6 = every day. HCWs who experienced burnout once a week or more were considered to have high burnout (Dolan et al., 2015).

#### 3.6.4 Job satisfaction

A validated single‐item derived from the Job Satisfaction Scale (JSS) was used to assess job satisfaction (Dolbier et al., 2005). We asked, “*Taking everything into consideration, how do you feel about your job as a whole?*”. The question was measured on a seven‐point Likert scale (extremely dissatisfied = 1 to extremely satisfied = 7), such that a higher score suggests increasing work satisfaction. HCWs who reported any form of dissatisfaction were considered to be “dissatisfied.”

#### 3.6.5 Covariates

The following covariates were considered: gender (male/female/others), age as a discrete variable, highest level of education (first degree/masters/PhD/fellowship), where first degree was obtained (Nigeria/Other African country/Outside Africa), profession (medical doctor/dentist/nurse/ medical lab scientist/nutritionist/physiotherapist/pharmacist/radiographer/optometrist/ occupational therapist/speech therapist/dental therapist/technician/others), marital status (single/married/cohabiting/separated/divorced/widowed), religion (Christianity/Islam/Others) and the state where the HCWs were born (e.g., Kano and Ekiti, which was classified into the six geopolitical zones in Nigeria [North Central, North East, North West, South East, South South and South West]); also the six geopolitical zones were dichotomized into two (North and South). Other details obtained from the HCWs include current employment status, that is, their employment status during the survey (employed/unemployed), the state where the HCWs currently practise (e.g., Lagos and Sokoto), work setting (teaching hospital, Federal Medical Centre, general hospital, primary health care, private facility, non‐governmental organization), the area where the HCWs resides (rural/urban), duration of practice as a health professional and the weekly number of work hours.

### 3.7 Data analysis

Data were downloaded in Microsoft Excel format from Survey Monkey. Data analysis was conducted using SPSS version 26. First, categorical variables such as gender and level of education were reported with frequency and percentage. Continuous and discrete variables such as age and weekly work hours were checked for normalcy using the Shapiro–Wilk test. Normally distributed data were reported with a mean (and standard deviation). Second, variables with a *p*‐value <0.25 were considered for multivariate analysis. But before then, the variance inflation factor (VIF) was used to check for multicollinearity between all these variables (i.e., VIF: ≥7.0). There was no evidence of multicollinearity. Finally, binary logistic regression was computed to identify the factors associated with migration intention among HCWs in Nigeria; the adjusted logistic regression analysis was performed at a 95% confidence interval. The fitness of the binary regression model was tested using the Hosmer–Lemeshow test, while the Nagelkerke *R* ^2^ test was used to assess the percentage of variance explained by the variables included in the regression model.

## 4 RESULTS

### 4.1 Characteristics of the HCWs

Most of the HCWs were females (55.4%), between the ages of 30 and 39 (38.5%), with a mean age of 38.1 ± 10.0 years (Table 1). Half (56.6%) only had a first degree, and about two in 10 (19.4%) had a master's degree. The majority of the HCWs studied in a federal university in Nigeria (75.3%). Most of the HCWs were doctors (31.0%) and nurses (29.6%), while 19.3% were physical/occupational therapists, 9.6% Pharmacists, 4.7% Medical Lab Scientist and other allied workers 5.8%. Most were born (47.3%) and currently practise (55.9%) in the South Western region of Nigeria, and about 9 in 10 of them (92.0%) were employed in a tertiary hospital (52.4%). The average duration of practice as a HCW was 12.0 ± 9.5 years (Table 2). About 19.9% reported daily burnout, and 6.6% were extremely dissatisfied with their job.

**TABLE 1:** Sociodemographic characteristics of the health workers (*N* = 513).

Variables	*N*	*n* (%)
Gender	513	
Female		284 (55.4%)
Male		229 (44.6%)
Age (years)	509	
20–29		115 (22.6%)
30–39		196 (38.5%)
40–49		121 (23.8%)
≥50		77 (15.1%)
Age mean (SD)	509	38.1 (10.0)
Highest level of education	511	
Diploma		28 (5.5%)
Fellow/PhD		38 (7.4%)
Masters		99 (19.4%)
Residency		57 (11.2%)
First degree		289 (56.6%)
First degree location	498	
Federal University in Nigeria		375 (75.3%)
Private Nigerian University		24 (4.8%)
State University in Nigeria		65 (13.1%)
University in other part of Africa		14 (2.8%)
University outside Africa		20 (4.0%)
Profession	513	
Doctor		159 (31.0%)
Nurse		152 (29.6%)
Physical therapist/OT		99 (19.3%)
Pharmacist		49 (9.6%)
Medical lab scientist		24 (4.7%)
Other allied professional		30 (5.8%)
Marital status	513	
Single/Never married		148 (28.8%)
Married/Cohabiting		343 (66.9%)
Ever married		22 (4.3%)
Ethnicity	511	
Yoruba		237 (46.4%)
Hausa		82 (16.0%)
Igbo		75 (14.7%)
Others		117 (22.9%)
Religion	509	
Christianity		357 (70.1%)
Islam		152 (29.9%)
Region born in	510	
North Central		61 (12.0%)
North East		49 (9.6%)
North West		82 (16.1%)
South East		40 (7.8%)
South South		37 (7.3%)
South West		241 (47.3%)

Abbreviation: OT, occupational therapist.

**TABLE 2:** Employment and work‐related characteristics of the health workers.

Variables	*N*	*n* (%)
Employment status	513	
Employed		472 (92.0%)
Unemployed		41 (8.0%)
Region of current practice	479	
North Central		61 (12.7%)
North East		43 (9.0%)
North West		67 (14.0%)
South East		12 (2.5%)
South South		28 (5.8%)
South West		268 (55.9%)
Work setting	483	
NGO/Others		72 (14.9%)
Private		64 (13.3%)
Primary		22 (4.6%)
Secondary		72 (14.9%)
Tertiary		253 (52.4%)
Duration of practice	507	
1–5		150 (29.6%)
6–10		126 (24.9%)
11–15		89 (17.6%)
16–20		55 (10.8%)
>20		87 (17.2%)
Duration of practice mean (SD)	507	12.0 (9.5)
Work hours per day	499	
1–8 h		290 (58.1%)
>8 h		209 (41.9%)
Work hours per day mean (SD)	499	9.3 (2.9)
Burnout	513	
Never		51 (9.9%)
A few times a year or less		81 (15.8%)
Once a month or less		32 (6.2%)
A few times a month		116 (22.6%)
Once a week		12 (2.3%)
A few times a week		119 (23.2%)
Every day		102 (19.9%)
Job satisfaction	513	
Extremely dissatisfied		34 (6.6%)
Dissatisfied		70 (13.6%)
Somewhat dissatisfied		100 (19.5%)
Neutral		101 (19.7%)
Somewhat satisfied		104 (20.3%)
Satisfied		93 (18.1%)
Extremely satisfied		11 (2.1%)

### 4.2 Migration intention

Two‐thirds (65.6%) had not migrated within Nigeria. However, internal migration has mostly occurred from North to North (13.4%; within the northern region), followed by South to South (8.8%; within the Southern region). Only 6.1% of HCWs migrated from North to Southern Nigeria and vice versa (Table 3). When asked about general migration intention (i.e., willingness to migrate as a non‐HCW), eight in ten (80.1%) of the HCWs were willing to migrate outside Nigeria. The five most common destinations include the United Kingdom (109 HCWs), Canada (92 HCWs), United States (82 HCWs), Australia (34 HCWs) and Saudi Arabia (23 HCWs). Approximately 70% of those willing to migrate indicated a willingness to stay back in Nigeria if relevant authorities address the major reasons responsible for migration intention.

**TABLE 3:** Migration intention and willingness to stay back.

Variables	*N*	*n* (%)
Intramigration experience	477	
No intramigration		313 (65.6%)
North to North		64 (13.4%)
North to South		29 (6.1%)
South to North		29 (6.1%)
South to South		42 (8.8%)
General migration intention	513	
Not at all		50 (9.7%)
Somewhat		100 (19.5%)
A little bit		84 (16.4%)
A lot		273 (53.2%)
Don't know		6 (1.2%)
Migration intention	513	
Yes		411 (80.1)
No		102 (19.9)
Job downgrading	389	
Yes		254 (65.3%)
No		135 (34.7%)
Election influence	387	
No, the election did not influence my decision to migrate		258 (66.7%)
Yes, because I am not satisfied with the election outcomes		102 (26.4%)
Yes, but I am satisfied with the election outcomes		27 (7.0%)
Countries to migrate to^a^		
United Kingdom		109
Canada		92
United States		82
Australia		34
Saudi Arabia		23
Others		68
Willingness to stay back with improve working and societal conditions	373	
Yes		260 (69.7%)
No		113 (0.3%)

^a^ Multiple responses.

Several reasons were reported to have instigated wanting to leave Nigeria, that is, the push factors. Several HCWs strongly agreed that poor salary (68%), poor economic conditions (66%) and lack of satisfaction with work environment (52%) were the major reasons for having the intention to migrate. Also, securing a better life for the family (66%), availability of modern equipment (64%) and health infrastructure (64%) were the most commonly reported pull factors (Table 4).

**TABLE 4:** Push and pull factors (*N* = 309).

Push factors	Strongly disagree	Disagree	Neutral	Agree	Strongly agree
Curiosity to travel and work abroad	37 (12%)	44 (14%)	85 (28%)	100 (32%)	43 (14%)
Working abroad is an opportunity to change lifestyle	10 (3%)	13 (4%)	41 (13%)	141 (46%)	104 (34%)
Lack of personal safety	9 (2%)	34 (11%)	58 (19%)	107 (35%)	101 (33%)
Poor salary/remuneration	8 (2.5%)	3 (1.0%)	20 (6.5%)	69 (22%)	209 (68%)
Lack of good health insurance	4 (1.3%)	9 (2.9%)	33 (10.7%)	113 (36.6%)	150 (48.5%)
Poor economic condition	3 (1.0%)	3 (1.0%)	18 (5.8%)	82 (27%)	203 (66%)
Fear of unemployment	19 (6.1%)	75 (24%)	83 (27%)	79 (26%)	53 (17%)
Political instability	9 (2.9%)	15 (4.9%)	51 (17%)	119 (39%)	115 (37%)
Insecurity and kidnapping for ransom	4 (1.3%)	19 (6.1%)	31 (10%)	102 (33%)	153 (50%)
Lack of opportunity for career development	7 (2.3%)	32 (10%)	46 (15%)	99 (32%)	125 (40%)
Lack of opportunity for professional advancement	5 (1.6%)	33 (11%)	55 (18%)	101 (33%)	115 (37%)
High workload and stress	5 (1.6%)	37 (12%)	64 (21%)	81 (26%)	122 (39%)
Lack of satisfaction with work environment and condition	4 (1.3%)	10 (3.2%)	27 (8.7%)	108 (35%)	160 (52%)
Lack of appreciation from supervisors	6 (1.9%)	40 (13%)	81 (26%)	96 (31%)	86 (28%)
Nepotism and tribalism in career advancement	14 (4.5%)	47 (15%)	77 (25%)	82 (27%)	89 (29%)
Low social status in the society	8 (2.6%)	53 (17%)	74 (24%)	108 (35%)	66 (21%)
Unsafe environment for family	3 (1.0%)	31 (10%)	42 (14%)	115 (37%)	118 (38%)
Lack of modern equipment	1 (0.3%)	10 (3.2%)	27 (8.7%)	115 (37%)	156 (50%)
Lack of adequate health infrastructure	2 (0.6%)	6 (1.9%)	16 (5.2%)	130 (42%)	155 (50%)
Pull factors					
High salary/remuneration	5 (1.6%)	2 (0.6%)	22 (7.1%)	118 (38%)	162 (52%)
Better economic activities and benefits	5 (1.6%)	2 (0.6%)	14 (4.5%)	119 (39%)	169 (55%)
Career development opportunities	4 (1.3%)	7 (2.3%)	11 (3.6%)	107 (35%)	180 (58%)
Better job opportunities	3 (1.0%)	3 (1.0%)	21 (6.8%)	125 (40%)	157 (51%)
Job security	3 (1.0%)	15 (4.9%)	69 (22%)	112 (36%)	110 (36%)
Less workload	22 (7.1%)	71 (23%)	112 (36%)	56 (18%)	48 (16%)
Securing a better future for my family	2 (0.6%)	3 (1.0%)	17 (5.5%)	82 (27%)	205 (66%)
Opportunity to become a permanent resident in a more developed country	3 (1.0%)	17 (5.5%)	62 (20%)	101 (33%)	126 (41%)
Job opportunities for family members	4 (1.3%)	12 (3.9%)	42 (14%)	129 (42%)	122 (39%)
Societal respect offered to health care workers	6 (1.9%)	16 (5.2%)	69 (22%)	114 (37%)	104 (34%)
Availability of modern equipment	4 (1.3%)	1 (0.3%)	13 (4.2%)	93 (30%)	198 (64%)
Availability of adequate health infrastructure	2 (0.6%)	2 (0.6%)	9 (2.9%)	98 (32%)	198 (64%)

### 4.3 Factors associated with migration intention

Age, duration of practice as an HCW, work hours, education, profession, marital status, ethnicity, employment status, burnout and job satisfaction were associated with migration intention in the crude regression model (*p* < 0.25; Table 5). Interestingly, migration intention was similar between HCWs in Southern and Northern regions. When the significant variables were fitted in a regression model (adjusted regression), three variables, namely, duration of practice, profession and burnout, were significantly associated with migration intention (Table 6). Working for a prolonged period of time as an HCW was associated with less willingness to migrate (AOR: 0.858; 95% CI: 0.782–0.941). Nurses were more likely to have migration intent than doctors (AOR: 3.866; 95% CI: 1.479–10.104); physiotherapists, pharmacists and medical lab scientists were insignificantly more likely to have migration intent than doctors. HCWs who reported no burnout were less likely to be willing to migrate outside Nigeria (AOR: 0.358; 95% CI: 0.192–0.665). Intramigration experience did not influence willingness to migrate outside Nigeria. The explanatory variables explained 27.5% of the migration intention variance. The model proved to fit (Hosmer and Lemeshow test [χ^2^: 5.774; *p*: 0.672]).

**TABLE 5:** Bivariate analysis between explanatory variables and migration intention among health workers in Nigeria.

Variables	Migration intention		Crude		*p*‐value
	No	Yes	Odds ratio	95% CI	
Age	44.2 (12.6)^a^	36.5 (8.6)^a^	0.929	0.908–0.950	<0.001
Duration of practice	18.5 (12.0)^a^	10.4 (7.9)^a^	0.919	0.898–0.941	<0.001
Work hours per day	8.9 (2.1)^a^	9.4 (3.1)^a^	1.081	0.986–1.185	0.096
Gender					
Female	44 (19.2)	185 (80.8)	1		
Male	58 (20.4)	226 (79.6)	1.079	0.697–1.671	0.733
Highest level of education					
Bachelor	48 (16.6)	241 (83.4)	1		
Diploma	3 (10.7)	25 (89.3)	1.660	0.482–5.718	0.422
Masters	26 (26.3)	73 (73.7)	0.559	0.324–0.964	0.036
Residency	10 (17.5)	47 (82.5)	0.936	0.442–1.981	0.863
Fellowship/PhD	15 (39.5)	23 (60.5)	0.305	0.149–0.628	0.001
First degree location					
Federal University in Nigeria	80 (21.3)	295 (78.7)	1		
State University in Nigeria	10 (15.4)	55 (84.6)	1.492	0.728–3.057	0.275
Private Nigerian University	5 (20.8)	19 (79.2)	1.031	0.373–2.845	0.954
University in other part of Africa	3 (21.4)	11 (78.6)	0.994	0.271–3.650	0.993
University outside Africa	2 (10.0)	18 (90.0)	2.441	0.555–10.739	0.238
Profession					
Medical doctor/Dentist	31 (19.5)	128 (80.5)	1		
Nurse	30 (19.7)	122 (80.3)	0.985	0.563–1.724	0.958
Physiotherapist/Occupational therapist	18 (18.2)	81 (81.8)	1.090	0.572–2.075	0.793
Pharmacist	15 (30.6)	34 (69.4)	0.549	0.266–1.131	0.104
Medical lab scientist	4 (16.7)	20 (83.3)	1.211	0.386–3.798	0.743
Others	4 (13.3)	26 (86.7)	1.574	0.512–4.841	0.429
Marital Status					
Single/Never married	21 (14.2)	127 (85.8)	1		
Married/Cohabiting	73 (21.3)	270 (78.7)	0.612	0.360–1.038	0.069
Separated/Divorced/Widowed	8 (36.4)	14 (63.6)	0.289	0.108–0.774	0.013
Ethnicity					
Yoruba	57 (24.1)	180 (75.9)	1		
Igbo	12 (16.0)	63 (84.0)	1.662	0.838–3.299	0.146
Hausa	16 (19.5)	66 (80.5)	1.306	0.701–2.433	0.400
Others	16 (13.7)	101 (86.3)	1.999	1.091–3.663	0.025
Religion					
Islam	29 (19.1)	123 (80.9)	1		
Christianity	71 (19.9)	286 (80.1)	0.950	0.587–1.536	0.833
Region born in					
North Central	11 (18.0)	50 (82.0)	1		
North East	8 (16.3)	41 (83.7)	1.127	0.415–3.065	0.814
North West	15 (18.3)	67 (81.7)	0.983	0.416–2.322	0.968
South East	7 (17.5)	33 (82.5)	1.037	0.365–2.948	0.945
South South	7 (18.9)	30 (81.1)	0.943	0.330–2.695	0.913
South West	53 (22.0)	188 (78.0)	0.780	0.380–1.604	0.500
Employment status					
Unemployed	97 (20.6)	37 (79.4)	1		
Employed	5 (12.2)	36 (87.8)	0.537	0.205–1.405	0.205
Region of current practice					
North Central	14 (23.0)	47 (77.0)	1		
North East	6 (14.0)	37 (86.0)	1.837	0.644–5243	0.256
North West	13 (19.4)	54 (80.6)	1.237	0.529–2.895	0.623
South East	2 (16.7)	10 (83.3)	1.489	0.291–7.611	0.632
South South	7 (25.0)	21 (75.0)	0.894	0.315–2.536	0.833
South West	56 (20.9)	212 (79.1)	1.128	0.580–2.194	0.723
Region of current practice^b^					
North	33 (19.3)	138 (80.7)	1		
South	65 (21.1)	243 (78.9)	0.894	0.560–1.428	0.639
Work setting					
Tertiary	44 (17.4)	209 (82.6)	1		
Secondary	14 (19.4)	58 (80.6)	0.872	0.447–1.701	0.688
Primary	4 (18.2)	18 (81.8)	0.947	0.306–2.936	0.925
Private facility	14 (21.9)	50 (78.1)	0.752	0.382–1.478	0.408
Non‐governmental organization/Others	20 (27.8)	52 (72.2)	0.547	0.298–1.007	0.053
Burnout					
Yes	28 (12.0)	205 (88.0)	1		
No	74 (26.4)	206 (73.6)	0.380	0.236–0.612	<0.001
Job satisfaction					
Satisfied	78 (25.2)	231 (74.8)	1		
Not satisfied	24 (11.8)	180 (88.2)	2.532	1.540–4.164	<0.001
Intramigration experience					
No	71 (22.7)	242 (77.3)	1		
Yes	26 (15.9)	138 (84.1)	1.557	0.949–2.556	0.080
Job downgrading					
Yes	254 (100.0)	254 (100.0)	–	–	–
No	135 (100.0)	135 (100.0)	–	–	–
Election influence					
No	258 (100.0)	258 (100.0)	–	–	–
Yes, not satisfied	102 (100.0)	102 (100.0)	–	–	–
Yes, satisfied	27 (100.0)	27 (100.0)	–	–	–
Willingness to stay back with improve working and societal conditions					
Yes	113 (100.0)	113 (100.0)	–	–	–
No	260 (100.0)	260 (100.0)	–	–	–

*Note*: migration intention signifies intention for international destinations.

^a^ Mean (SD).

^b^ Region of practice dichotomized.

**TABLE 6:** Multivariate analysis between explanatory variables and migration intention among health workers in Nigeria.

Variables	Odds ratio	95%	CI	*p*‐value
Age	1.044	0.957	1.138	0.335
Duration of practice	0.858	0.782	0.941	**0.001**
Work hours per day	1.043	0.924	1.177	0.498
Education				
Bachelor	1			
Diploma	1.200	0.223	6.458	0.832
Masters	1.405	0.686	2.875	0.353
Residency	1.100	0.392	3.088	0.856
Fellowship/PhD	1.045	0.380	2.869	0.933
First degree location				
Federal University in Nigeria	1			
State University in Nigeria	1.287	0.532	3.117	0.575
Private Nigerian University	0.616	0.199	1.909	0.401
University in other part of Africa	0.414	0.095	1.812	0.242
University outside Africa	1.536	0.306	7.717	0.602
Profession				
Medical doctor/Dentist	1			
Nurse	3.866	1.479	10.104	**0.006**
Physiotherapist/Occupational therapist	1.679	0.653	4.320	0.282
Pharmacist	1.082	0.403	2.903	0.876
Medical lab scientist	1.847	0.408	8.352	0.426
Others	2.284	0.528	9.885	0.269
Marital status				
Single/Never Married	1			
Married/Cohabiting	1.576	0.699	3.556	0.273
Separated/Divorced/Widowed	1.236	0.313	4.873	0.763
Ethnicity				
Yoruba	1			
Igbo	1.173	0.484	2.844	0.724
Hausa	0.504	0.197	1.291	0.153
Others	1.089	0.480	2.466	0.839
Employment status				
Unemployed	1			
Employed	0.903	0.087	9.332	0.932
Work setting				
Tertiary	1			
Secondary	1.018	0.453	2.289	0.965
Primary	0.814	0.206	3.214	0.769
Private facility	0.878	0.343	2.249	0.786
Non‐governmental organization/Others	0.755	0.332	1.719	0.503
Burnout				
Yes	1			
No	0.358	0.192	0.665	**0.001**
Job satisfaction				
Satisfied	1			
Not satisfied	1.701	0.904	3.201	0.100
Intramigration experience				
No	1			
Yes	1.179	0.608	2.287	0.626

*Note*: Migration intention signifies intention for international destinations. Bolded *p*‐values are significant.

## 5 DISCUSSION

This study investigates emigration intentions among HCWs in Nigeria. The study has shown that the emigration intention rate of 80.1% is higher than what was reported in earlier studies (Adebayo & Akinyemi, 2022; Akinwumi et al., 2022; Alabi & Olajide, 2023; Onah et al., 2022). There are a few plausible reasons for these differences in emigration intentions between studies conducted before the 2023 elections and the current one. First, a lot has changed since February after the 2023 national elections. As can be gleaned from Table 3, about one‐third of the respondents said that the outcome of the 2023 elections partly or wholly influenced their decision to emigrate. This could be attributed to the dissatisfaction with the outcome of the 2023 presidential elections, where the winner is perceived to have rigged his way to success. As Alabi and Olajide (2023; p. 81) noted: “Many young people alleged that the electoral process was not free and fair and that political thugs openly intimidated perceived opponents and stopped them from voting. This was followed by expressions of hopelessness on social media and the consequent trending of #japa on Twitter.”

Second, fuel subsidies were removed in mid‐2023 without any serious prior notice or increase in the minimum wage. As of mid‐September 2023, one US dollar was exchanged for over 800 Nigerian Naira. Exchange rates may affect the decision to emigrate from one place to another (Keita, 2016). The finding that most HCWs preferred to emigrate to the United Kingdom can be explained by two factors. One, the United Kingdom recognizes Nigerian medical certificates. Hence, medical doctors practising in Nigeria can continue their job in the United Kingdom. Two, the British Pound is one of the strongest in the world against the Nigerian Naira (Keita, 2016).

Regarding push and pull factors, the findings of this study show that the leading push factor is poor remuneration – indicated by over two‐thirds of the participants, followed by poor economic conditions (66%) and lack of satisfaction with the work environment (52%). A major reason for this may be connected with comparing their earnings in Nigeria to what their counterparts in the West earn, thereby suggesting the urgency to leave the country and the refusal to cope (Liu, 2024). The leading pull factors are the ease of securing a better for their family (66%), availability of modern equipment (64%) and adequate health infrastructure (64%). Regarding a better life, it is often believed that Nigeria is unpredictable and volatile. Hence, people want to emigrate to avoid the mental burden of an insecure future and uncertainty. For example, due to incessant lecturers' strikes, medical students are unsure when they will complete their programme. In addition, federal government workers are uncertain of the date on which their next salary will be paid. In the West, however, a more straightforward career path is visible, and one can plan for their future. Moreover, the economic system is reliable, and workers know when they will receive payment for their work.

The study's findings on the influence of duration of practice, profession and burnout seem interesting at the multivariate level. Our finding on the association between burnout and emigration intention supports the early research of Győrffy et al. (2018). It suggests that the intention to emigrate may transcend financial reasons espoused by economic theories of migration. It shows how unpleasant experiences at work may push people to want to emigrate (Yakubu et al., 2023). The finding that HCWs who have practised for a prolonged period had a lower likelihood of emigration intention is interesting and yet unexpected. Although years of experience is expected to be correlated with age, and since young people migrate and have migration intentions more than older people (Alabi & Olajide, 2023), it makes sense to find that HCWs with lower years of work experience have higher emigration intentions. However, it is logical to expect that HCWs who have worked for a longer period and must have had more unpleasant experiences will have higher odds of emigration intention than HCWs who are new on the job. However, it could be that HCWs with more experience have had financial and social establishments in Nigeria, making it tougher for them to want to emigrate and start life afresh in another country. Our finding that nurses had higher odds of emigration intention than medical doctors supports the earlier study of Yakubu et al. (2023). A possible reason for this is that nurses are misrepresented in Nigeria; they are often considered inferior to doctors. In the media and movies, they are portrayed as support staff to medical doctors. Hence, their pay is less than that of doctors despite their sacrifice to Nigeria's health sector (Azubuike, 2023).

In Nigeria, the intention of HCWs to emigrate is not unconnected to the global economic and social imbalance. Evidence of such imbalance includes wage differentials, availability of medical equipment that facilitates the jobs of HCWs, work environment in broad form and predictability of the political and social system. These factors operate differently between Nigeria and Western countries, where most HCWs intend to emigrate. In Nigeria, modern medical facilities are not available to HCWs to do their job; the work environment is unfriendly as HCWs are overburdened by many patients, leaving some of them to work many hours without rest. Importantly, the Nigerian system is less predictable compared to the systems in the United Kingdom, United States, Canada and Australia.

### 5.1 Limitations

This study conducted a national survey on migration among HCWs in Nigeria, which is a major strength compared to similar related studies. However, there are some limitations to our study. This study was an online survey among HCWs in Nigeria, and there is a possibility that some of the responses were not from HCWs, which may have confounded our findings. Also, using a cross‐sectional study design and a non‐randomized sampling technique does not permit the generalization of our results, and we cannot claim causality. Additionally, the data obtained cannot be considered to be a national representation of HCWs' migration intention in Nigeria because most of the respondents practise in states within the Southern region of the country.

Furthermore, we did not consider HCWs' migration intention within Nigeria, which is equally important. Finally, the intention of HCWs is not synonymous with actual migration; therefore, our findings may not portray the migration issues around HCWs in Nigeria.

## 6 CONCLUSIONS

Many HCWs in Nigeria appear to have emigration intent, especially the nurses. The departure of HCWs in Nigeria may be associated with increasing burnout for the available HCWs, which may influence their willingness to leave the country. The Nigerian government may want to explore strategies to reverse the emigration intent of the HCWs in Nigeria. Specifically, new policies that will deter migration intention and actual migration are urgently needed to curb the current brain drain in the Nigerian health care system. Based on the major push factors reported by the HCWs, we recommend policies that would increase HCWs' salary and overall economic condition, as well as policies that will ensure that the primary, secondary and tertiary health facilities are well‐equipped and up to international standards.

## AUTHOR CONTRIBUTIONS

OAB and TAA conceived the research idea. All authors contributed to the research design. OAB, TAA, MAA, AA and SSO ensured data collection. Data analysis was conducted by OAB and verified TAB, SSO and MAA. OAB and TAA drafted the manuscript, and all authors critically revised the manuscript. OAA and FAB supervised the entire review process.

## FUNDING INFORMATION

This research received no specific grant from any funding agency in the public, commercial or not‐for‐profit sectors.

## CONFLICT OF INTEREST STATEMENT

The authors certify that they have no affiliations with or involvement in any organization or entity with any financial interest or non‐financial interest in the subject matter or materials discussed in this manuscript.

## ETHICS STATEMENT

The study protocol was approved by the Sokoto State Ministry of Health (SKHREC/019/2023). The study was conducted in line with the International Declaration of Helsinki’s principles and guidelines. All participants were informed of the study details before consent was provided.

## STATISTICS GRADING

Oluwaseun Abdulganiyu Badru is the statistician. Appropriate statistical tests were selected, and the data were interpreted correctly.

## Data Availability

The data that support the findings of this study are available on request from the corresponding author. The data are not publicly available due to privacy or ethical restrictions.
